# Enhanced Sentinel Surveillance System for COVID-19 Outbreak Prediction in a Large European Dialysis Clinics Network

**DOI:** 10.3390/ijerph18189739

**Published:** 2021-09-16

**Authors:** Francesco Bellocchio, Paola Carioni, Caterina Lonati, Mario Garbelli, Francisco Martínez-Martínez, Stefano Stuard, Luca Neri

**Affiliations:** 1 Fresenius Medical Care Italia SpA, Palazzo Pignano, 26020 Lombardia, Italy; francesco.bellocchio@fmc-ag.com (F.B.); paola.carioni@fmc-ag.com (P.C.); mario.garbelli@fmc-ag.com (M.G.); 2 Center for Preclinical Research, Fondazione IRCCS Ca’ Granda Ospedale Maggiore Policlinico, 20122 Milan, Italy; caterina.lonati@gmail.com; 3 Santa Barbara Smart Health S. L., Parc Cientific Universitat id Valencia, Carrer del Catedràtic Agustín Escardino Benlloch, 9, 46980 Paterna, Spain; francisco.martinezmartinez@fmc-ag.com; 4 Fresenius Medical Care Deutschland GmbH, 61352 Bad Homburg, Germany; Stefano.stuard@fmc-ag.com

**Keywords:** SARS-CoV-2, COVID-19, sentinel surveillance system, outbreak prediction, machine learning, artificial intelligence

## Abstract

Accurate predictions of COVID-19 epidemic dynamics may enable timely organizational interventions in high-risk regions. We exploited the interconnection of the Fresenius Medical Care (FMC) European dialysis clinic network to develop a sentinel surveillance system for outbreak prediction. We developed an artificial intelligence-based model considering the information related to all clinics belonging to the European Nephrocare Network. The prediction tool provides risk scores of the occurrence of a COVID-19 outbreak in each dialysis center within a 2-week forecasting horizon. The model input variables include information related to the epidemic status and trends in clinical practice patterns of the target clinic, regional epidemic metrics, and the distance-weighted risk estimates of adjacent dialysis units. On the validation dates, there were 30 (5.09%), 39 (6.52%), and 218 (36.03%) clinics with two or more patients with COVID-19 infection during the 2-week prediction window. The performance of the model was suitable in all testing windows: AUC = 0.77, 0.80, and 0.81, respectively. The occurrence of new cases in a clinic propagates distance-weighted risk estimates to proximal dialysis units. Our machine learning sentinel surveillance system may allow for a prompt risk assessment and timely response to COVID-19 surges throughout networked European clinics.

## 1. Introduction

Due to its unique characteristics, the Severe Acute Respiratory Syndrome Coronavirus 2 (SARS-CoV-2) pandemic has posed unprecedented challenges to clinics providing life-saving services to patients suffering from chronic illnesses, including chronic kidney disease (CKD). In fact, non-specific clinical manifestations of Coronavirus disease 2019 (COVID-19) [1] as well as the viral transmission from asymptomatic or pre-symptomatic individuals [2,3,4] make the early recognition of newly infected cases extremely difficult. Moreover, the occurrence of superspreading events (SSEV), during which few individuals are able to infect many people [5], hampers infection control measures [6,7].

Social distancing, preventive quarantine, and the isolation of infected subjects still represents the most effective means to reduce the risk of SARS-CoV-2 human-to-human transmission [8,9]. However, patients with end-stage kidney disease (ESKD) need to undergo in-center dialysis three times per week for 4 h per session, which makes physical distancing more difficult to achieve due to repeated, prolonged interactions with other patients and healthcare staff [10,11,12,13]. Unfortunately, ESKD individuals also show a higher risk of complications following SARS-CoV-2 infection due to weakened immune response [14,15,16,17] and to the occurrence of many of the risk factors commonly associated with development of severe COVID-19 [18,19], including older age and comorbidities [20,21]. Moreover, because of compromised host immunity, a vaccine may not exhibit the same efficacy on hemodialysis patients as it does in immunocompetent individuals [13]. 

Therefore, the reduction of the contagion risk within dialysis clinics while preserving clinical operations is a key challenge for healthcare systems during this pandemic. To help anticipate local epidemic dynamics and adjust non-pharmacological interventions to the changing background of infection risk, we sought to develop an advanced sentinel surveillance system supported by a machine learning (ML) prediction model, where the occurrence of COVID-19 cases in a clinic propagates distance-weighted risk estimates to adjacent dialysis units. The present study describes the derivation and validation of the prediction model, as well as the strategies adopted to monitor its performance throughout the pandemic period.

## 2. Materials and Methods

### 2.1. Design and Setting

All dialysis clinics belonging to the Fresenius Medical Care (FMC) European Nephrocare Network confer clinical data to a centralized data-repository, namely the European Clinical Database (EuCliD^®^, Fresenius Medical Care, Deutschland GmbH, Vaiano Cremasco, Italy) [22,23]. Since April 2020, all SARS-CoV-2 infections (suspected and confirmed cases as well as initial symptoms), diagnostic procedures, and clinical endpoints are reported in the treatment incident report (TIR) module in EuCLiD^®^. We used aggregated data abstracted from the TIR, open source data describing epidemic dynamics in European countries, as well as aggregated data on biochemical assays prescriptions and results to estimate outbreak risk in dialysis clinics belonging to the FMC European Nephrocare Network. 

### 2.2. Outcome Variable

**Figure 1:**
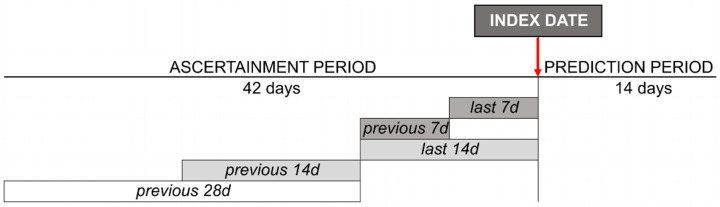
Study design: Reference timeframe for data collection/calculation is shown.

The model forecasts the risk of a COVID-19 outbreak in each dialysis clinic in a 2-week horizon. Clinic outbreak is defined as the occurrence of two or more COVID-19-confirmed cases in a given clinic. Therefore, for each clinic registered in the Nephrocare network, the model estimates the probability of COVID-19 outbreak (2 or more PCR confirmed cases within a 2-week horizon) as a function of a vector of input variables. Study design is represented in Figure 1.

For illustrative purposes, we established 3 risk categories: (1) low (L), when outbreak risk is less than or equal to 1.5%; (2) medium (M), risk greater than 1.5% and less than or equal to 12.5%; (3) high (H), if risk is greater than 12.5%. For this purpose, the action threshold defining the low risk class has been chosen to select a subpopulation of clinics where the risk of outbreak is very small so that non-pharmacological interventions to prevent the spread of COVID-19 can be temporarily and partially mitigated. In this context, a costly error would be to assign to the Low Risk class a clinic which will experience an outbreak in the following two weeks. Such threshold would be useful when a sufficiently large share of clinics (i.e., P(Class = L)) could be found, so that P(Class = L|Outbreak = No) is high and P(Outbreak = Yes|Class = L) is, conversely, very small. On the other hand of the spectrum, we selected a more specific action threshold, which defines a High-Risk Class of clinics. In this risk group, additional non-pharmacological intervention should be initiated including, for example, the formal testing of temperature and thorough physical examination administered to each patient before entering the clinic or even periodical screening test (i.e., once-weekly). Since the intervention would require intensive resources, may be constraint by procurement difficulties, and would unduly overburden patients with unnecessary testing, the High Risk threshold should ideally define a group where P(Outbreak = Yes|Class = H) is high and both P(Class = H|Outbreak = No) and P(Outbreak = Yes|Class ≠ H) are low. It is important to remark that the choice and number of the action thresholds depends on the intended use of the risk score, the set of interventions available to the organization, the price cost of each intervention, and ultimately by the value function ranking the desirability/undesirability of different health outcomes. Therefore, the thresholds presented in this paper should not be considered generalizable per se: different institutions may choose different thresholds (or no thresholds at all) depending on the availability, cost, and expected outcomes of COVID-19-related interventions (i.e., email alerts to medical directors, shipments of medical equipment such as face masks or diagnostics kits, delivery of health education modules, PCR screening, etc.,). Therefore, the problem is not diagnostic in nature, yet reduces to optimal ranking (and longitudinal stability of such ranking of risk) in order to efficiently allocate limited resources and minimize risk for the patients throughout a continuously changing epidemic landscape.

### 2.3. Input Variables

The model is computed using aggregated data provided by all the dialysis centers (min: 545; max: 611) located in one of the 23 countries of the FMC European Nephrocare Network. The final model incorporates 74 variables belonging to one of the following categories (Appendix A):

1. Open Source Data [24];
2. Epidemic status in the clinical country/region (prefix: RG): 15 parameters;
3. Aggregated Data abstracted from EuCLiD^®^:
  - Epidemic status in the target clinic (prefix: CL): 5 variables;
  - Distance-weighted information of the adjacent clinics (prefix: CLS); 5 variables. Adjacent clinics were defined as the 3 centers with shorter distance in terms of both latitude and longitude to the target clinic. Measures of the adjacent clinics, including cases and trends, were computed as the average value weighted for the inverse of the distance to the target clinic;
  - Other parameters related to the target clinic (prefix: CL): 49 parameters.

As detailed in Appendix A, each variable can be calculated/collected over different timeframes of the ascertainment period, i.e., the last 7 days (d), previous 7 d, last 14 d, previous 14 d, and previous 28 d.

### 2.4. Statistical Analysis

#### 2.4.1. Model Derivation

We used XG Boost, a scalable ML system for tree boosting [25]. We used the available open source package [26] for Python, Version 3.7.4 (Python Software Foundation, Delaware, DE, United States) [27]. 

The first release of the model was trained using data related to 1st April 2020 (training dataset index date), while the second and the third versions were derived using data related to 15th July 2020 and 1st November 2020, respectively. We considered all the clinics delivering services to at least one patient on the index date as well as over the week before index date.

#### 2.4.2. Model Accuracy and Feature Importance

Prediction accuracy of each release was tested every first and fifteenth day (validation dataset index dates). Therefore, development and validation datasets can include the same set of clinics/patients every two weeks.

To evaluate model performance, we measured the area under the curve (AUC) of the receiver operating characteristics (ROC) curve in the testing datasets [28] using Python, Version 3.7.4 (Python Software Foundation, Delaware, DE, United States) [27]. The AUC provides an aggregate measure of performance as the ROC curve plots the true positive rate (TPR) against the false positive rate (FPR) at all classification thresholds. Model discrimination ability over time was monitored by visual inspection of AUC trends. For illustrative purposes, we also reported the classification performance in terms of P(Outbreak|Class) (i.e., probability of outbreak (Yes/No) given the assigned risk class (L/M/H)) and P(Class|Outbreak) (i.e., probability of the assigned risk class given the outbreak) for the two action-thresholds chosen (0.015 and 0.125). In order to calculate P(Outbreak|Class) and P(Class|Outbreak) we artificially treated our problem as a binary decision for each threshold. We computed average probability values across the whole study period.

Feature importance was computed using the SHapley Additive exPlanations (SHAP) method [29]. This analysis enables intuitive model explainability via an accurate and efficient estimation of the contribution to risk of each input variable.

#### 2.4.3. Descriptive Statistics

For both the training and validation datasets, we analyzed the number of active clinics, frequency and incidence of a COVID-19 outbreak, the distribution of clinics in each prediction level of risk (low, medium, high), as well as the relative risk compared to clinics in low-risk groups with Python, Version 3.7.4.

## 3. Results

### 3.1. Dialysis Clinic Characteristics

Model version 1, 2, and 3 were trained using a dataset related to 1st April 2020, 15th July 2020, and 1st November 2020, respectively. On these dates, active clinics were 589, 597, 603, while 34 (5.77%), 44 (7.37%), and 233 (38.64%) clinics had two or more patients with COVID-19 infection in the fortnight after the index date.

**Figure 2:**
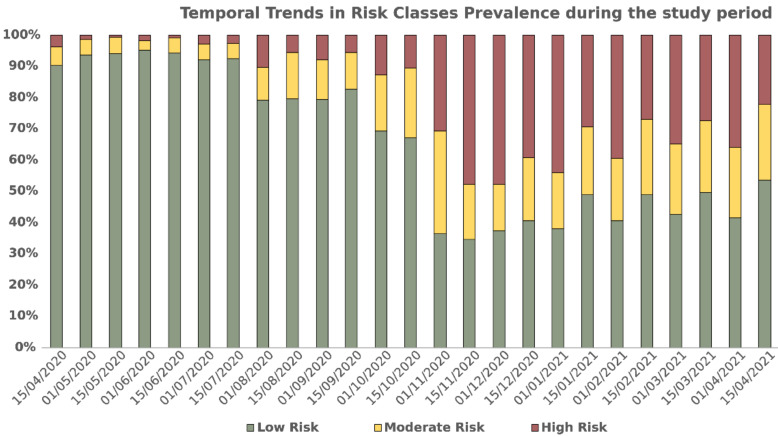
Number of dialysis clinics at the validation dates. Colors denote risk categories: Red, high > 12.5%; Yellow, medium 1.5% < x ≤ 12.5%; Green, low ≤ 1.5%.

The surveillance system stratifies clinics by their risk of new local outbreak within two weeks. To facilitate the interpretation of the results, we established three risk categories: (1) Low, when outbreak risk is less than or equal to 1.5%; (2) Medium, risk greater than 1.5% and less than or equal to 12.5%; (3) High, if risk is greater than 12.5%. Risk thresholds depend both on the incidence of pandemic and on the ability of any given clinic to implement containment measures. Figure 2 reports the share of active dialysis clinics in different risk classes at each testing date.

**Figure 3:**
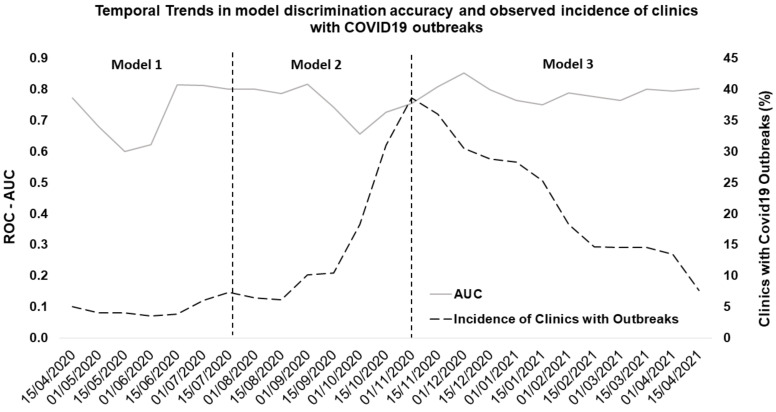
Model Performance and Incidence of Clinics with Outbreaks: the plot reports data related to the 1 year observation period.

The actual outbreak incidence in the dialysis clinics during the validation period is reported in Figure 3. 

### 3.2. Model Performance

**Figure 4:**
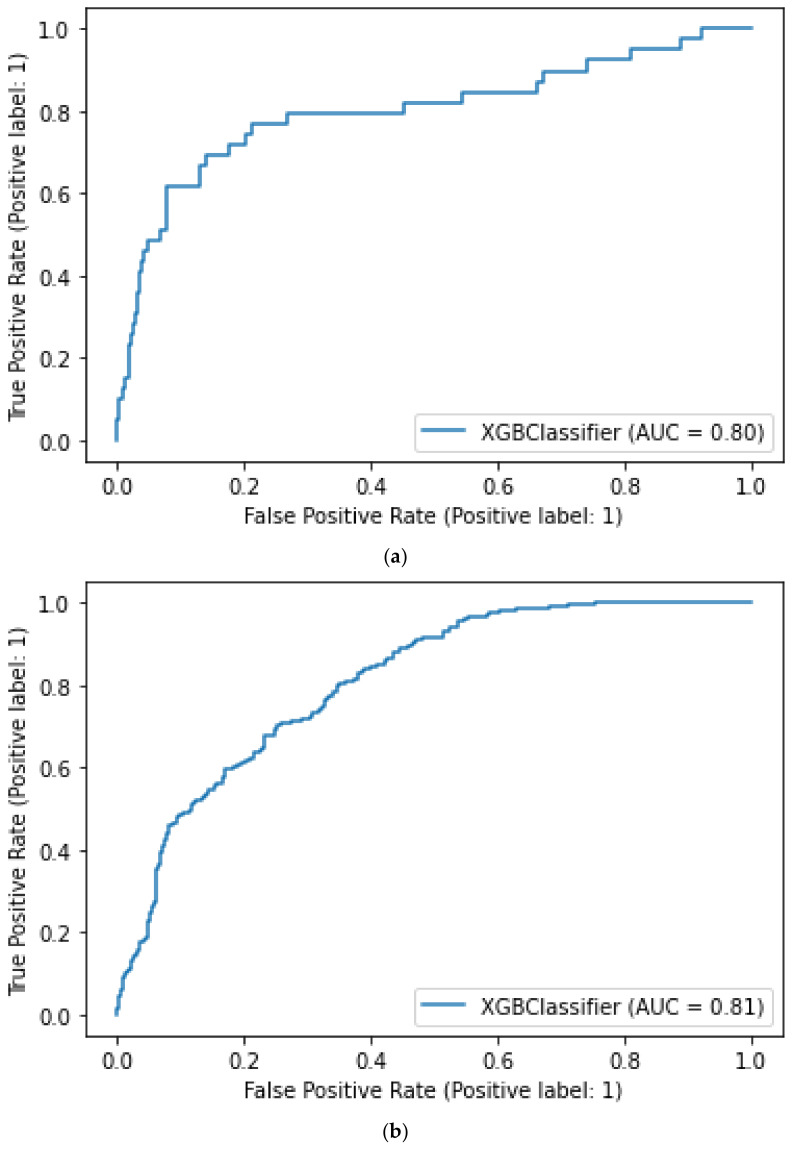

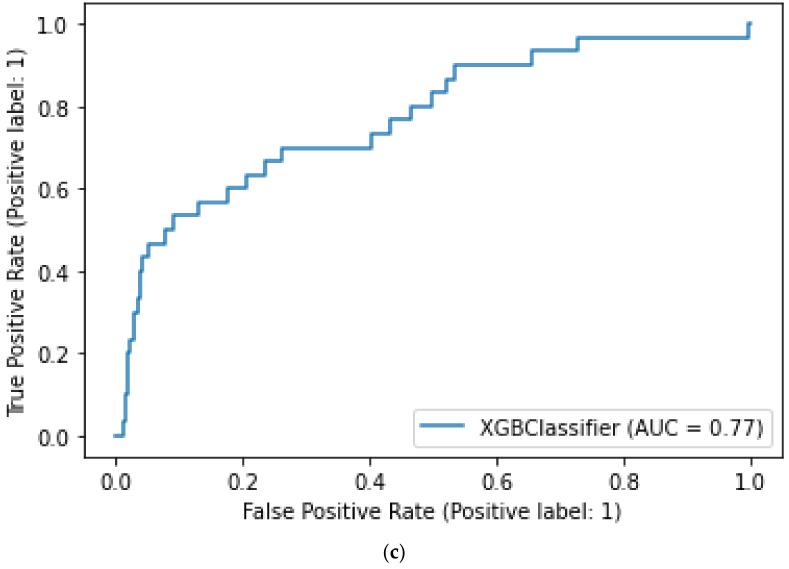
Panel (**a**–**c**) respectively contain the ROC-AUC plot related to Model 1, Model 2, and Model 3 evaluated on the following dates: 15 April 2020, 1 August 2020, and 15 November 2020.

All versions of the model showed a good performance over the validation period. Figure 3 shows trends in AUC values of the three model versions over a 1-year observation period. Variability in prediction accuracy decreased as retraining was applied: version 1’s average AUC was 0.73 (95% CI 0.55–0.91), AUC of version 2 was 0.75 (95% CI 0.65–0.86), while version 3 had a more stable performance with an average AUC of 0.79 (0.74–0.85). The ROC-AUC diagram for the three model versions have been reported in Figure 4.

**Figure 5:**
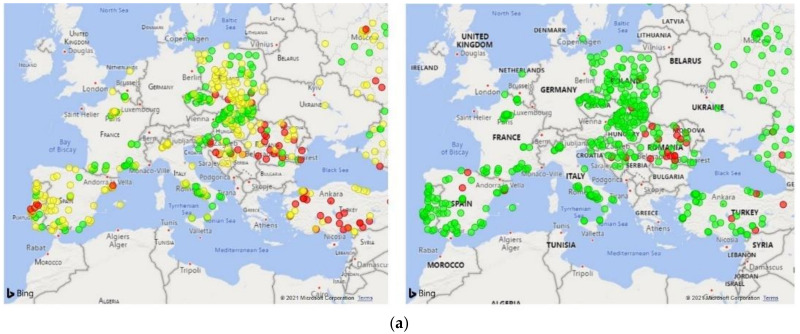

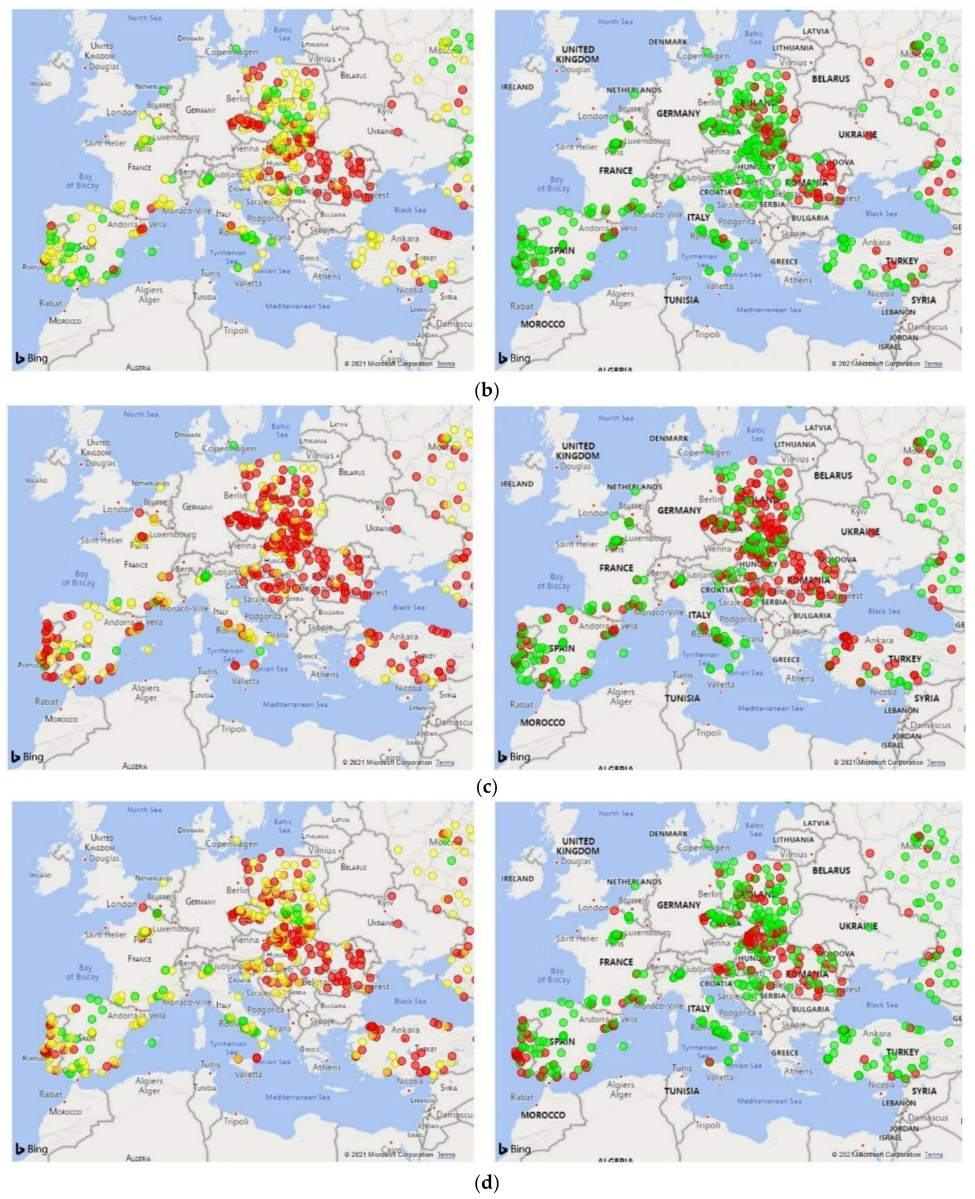
COVID-19 outbreak risk mapping in European clinics of the Nephrocare network. Geographical risk maps were built considering epidemic data related to the following exemplary dates: (**a**) 2 August 2020, (**b**) 4 October 2020, (**c**) 1 November 2020, and (**d**) 3 January 2020. Panels on the left show clinic clusters according to the risk of a COVID-19 outbreak occurrence within 2 weeks: Red circles: risk > 12.5%; Yellow, 1.5% < risk ≤ 12.5%; Green, risk ≤ 1.5%. Panels on the right report the actual incidence of COVID-19 outbreaks in the forecasting period.

In order to demonstrate the potential use of the model, we geographically mapped the risk on a few exemplary dates, i.e., the 2 August 2020, 4 October 2020, 1 November 2020, and 3 January 2020 (Figure 5). The graphical representation visually highlights clinic clusters according to the risk of a COVID-19 outbreak occurrence within 2 weeks (Figure 5, left panels, colored circles denote the low, medium, and high-risk categories). There was substantial correlation between the predicted risk (Figure 5, left panels) and the actual outcome (Figure 5, right panels) on all of the validation dates. 

Table 1 and Table 2 report the classification performance in terms of P(Outbreak|Class) (i.e., probability of outbreak (Yes/No) given the assigned risk class (L/M/H)) and P(Class|Outbreak) (i.e., probability of the assigned risk class given the outbreak) for the two action-thresholds chosen (0.015 and 0.125). In order to calculate P(Outbreak|Class) and P(Class|Outbreak), we artificially treated our problem as a binary decision for each threshold. We computed average probability values across the whole study period.

Overall, the risk score was strongly associated with the likelihood of COVID-19 outbreak, as demonstrated by the relative risk of outcome occurrence in the three risk classes over the study period (Table 3). 

### 3.3. Model Feature Importance

**Figure 6:**
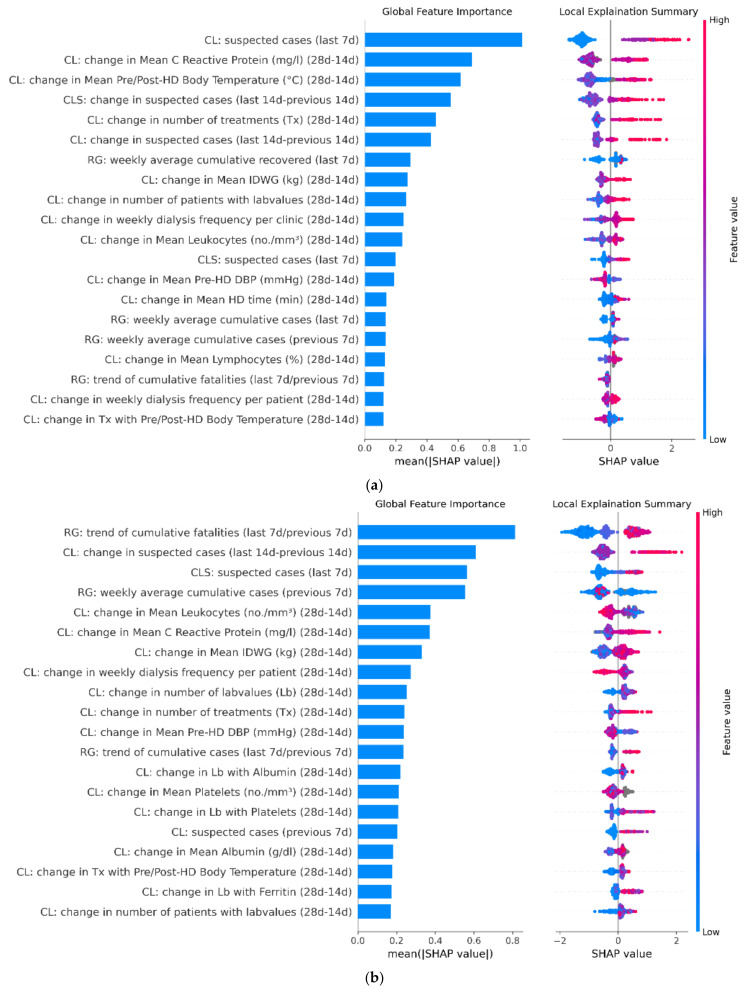

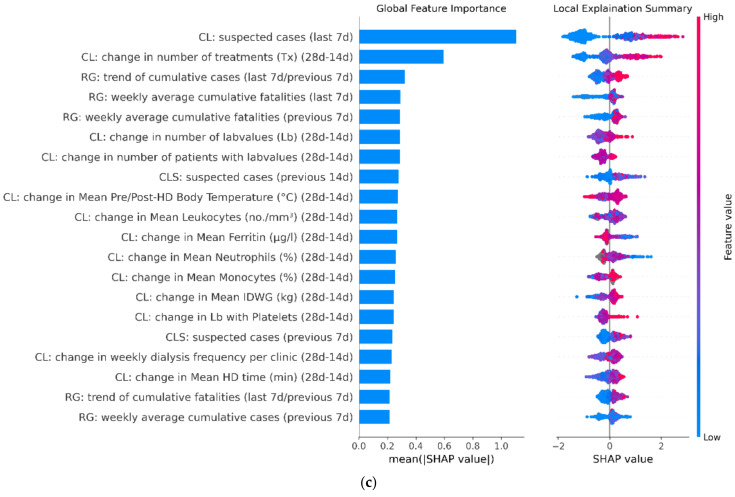
Panel (**a**–**c**) respectively contain the Shapley additive explanations (SHAP) related to Model 1, Model 2, and Model 3 evaluated on the following dates: 15 April 2020, 1 August 2020, and 15 November 2020. SHAP plots show relative feature importance. The blue bar represents overall SHAP values for each variable and are interpreted as relative importance of each variable to risk estimates. On the right side, SHAP values show the direction of association between predictor and risk estimates. Each dot represents one individual clinic from the test dataset. Higher values of the predictors are represented in red color; lower values of the predictors are represented in blue color. The X axis represents the impact of variables on risk in terms of SHAP values. Red color in correspondence with positive values suggests direct correlations between risk factors and the occurrence of COVID-19 outbreak, while red color in the region of negative SHAP values suggests inverse correlation.

Feature analysis investigated the impact of each variable on model output (Figure 6). Although there are some differences among the model versions, overall, the most important variables are related to the epidemic dynamics in the clinic in the period immediately preceding the index date for risk evaluation. Regional data on the number of COVID-19 cases and deaths were likewise ranked high. The number of COVID-19 cases in adjacent clinics resulted in the top predictor list of all three model versions. Of note, variables routinely measured in clinical practice, including changes in CRP and blood white cell count over the observation period, were also strongly associated with outbreak risk.

## 4. Discussion

The present study describes the development and validation of a novel sentinel surveillance system allowing for the prompt risk assessment of a COVID-19 outbreak in a large European network of dialysis clinics over a 2-week forecasting horizon. The model had a stable accuracy over time and was able to consistently discriminate outbreak risk in dialysis units across all European countries at every stage of the current pandemic, i.e., during epidemic growth and decay phases. The design of our ML prediction model enables administrators and developers to quickly retrain this tool in case the visual inspection of AUC values over time suggests a trend toward a decrease in its discrimination ability. 

Nosocomial transmission has greatly contributed to an increase in the global burden of COVID-19 pandemic by extremely affecting the capacity of the health system, not only to provide medical support to patients, but also to protect healthcare professionals [30,31]. Dialysis centers are particularly vulnerable to outbreak development [11,12,32] in that mitigation strategies are not entirely feasible due to the necessity of in-person encounters to provide a life-saving treatment such as hemodialysis [11]. Considering the peculiar frailty of ESKD patients, all scientific nephrology societies have provided guidance on COVID-19 transmission prevention in dialysis facilities [33,34,35]. In this regard, surveillance and early contagion detection are essential to reduce the risk of local outbreaks developing into epidemics.

Clinics of the FMC European Nephrocare Network have implemented multiple non-pharmacological interventions to limit viral spreading among the CKD community, including stringent hygiene procedures, social distancing, and the identification and isolation of suspected cases. In addition, dialysis facilities have established recording pathways to report any infection event in the EuCliD^®^ TIR System. Such data are used to monitor the effectiveness of non-pharmacological intervention and to detect high-risk patients needing special attention [36,37,38]. 

One important feature of our modeling strategy entailed the combined use of open source and clinical data collected in standard clinical practice. In fact, we exploited the interconnection of the European Nephrocare clinics to augment background epidemic data with a surveillance system based on incident reports and practice pattern variation at each dialysis unit. Information about local epidemic status in a given clinic was then propagated through distance-weighting metrics to the surrounding facilities. An ML method was used to integrate all information into a summary score metric. Remarkably, variables related to the epidemic dynamics in the clinic and to the regional epidemic status, as well as to the risk proxies propagated from adjacent clinics, were all important predictors of outbreak occurrence. Such an approach is particularly relevant because it enabled us to capture local disease spread beyond the registry data compiled for the general population, which does not capture the heterogeneity of viral transmission in a setting where frequent and multiple human interactions necessarily occur. Indeed, as the basic reproduction index (R0) is a function of both the transmissibility of a disease and the contact patterns that underlie transmission [39], the regional/provincial R0 cannot be translated in dialysis facilities in that ESKD patients’ biological and socio-behavioral factors significantly differ from those of the general population [40]. The occurrence of SSEVs further complicates the picture, making generalizations of regional epidemic trends that are not entirely appropriate for the reliable prediction of viral spreading in healthcare settings [41,42]. 

The interconnection of the FMC network allows for the collection and subsequent central integration of a bulk of information provided by facilities distributed throughout European countries. This particular setting offers the advantage to perform the real-time monitoring of sentinel sensors that are likely to provide timely and accurate indications of epidemic activity [43], while considering the heterogeneity underlying transmission dynamics. Sentinel surveillance in outpatient settings was previously shown to provide a robust approach to oversee SARS-CoV-2 spreading [44]. In general, the monitoring of community transmission in nodes distributed across different regions was reported to ensure efficient disease detection in networked populations [45]. It is important to highlight that the analytic strategy adopted in this study is general and can be applied to any epidemic communicable disease, as all naturally occurring, clustering units where social promiscuity, density, and duration of interactions are substantially different compared to the general population. Henceforth, this method may be applied to social contexts with a high risk of outbreak generation, including schools, hospitals, and workplaces from which the provided infection data are promptly captured and conferred to a central database, even in aggregated form. Monitoring of the pandemic situation within the network allows for the timely implementation of infection control procedures in the adjacent networked unit and efficiently anticipates resource needs. 

Finally, variable importance analysis has indicated that trends in clinical practice patterns are among the top predictors. This observation indicates that the tracking of physicians’ prescription behavior can provide valuable information to assess epidemic dynamics also during explosive growth, when surveillance and laboratory resources are limited and COVID-19 cases may be recorded with some delay due to the emergency situation [46].

## 5. Conclusions

Our sentinel surveillance system allows for a prompt risk assessment and timely response to the challenges posed by the COVID-19 epidemic throughout FMC European dialysis clinics. This tool can have significant implications for public health practice in that it represents a robust strategy to assess the level of community transmission of COVID-19 and to guide the selection and implementation of mitigation measures. The same framework can be applied in other networked settings, such as healthcare facilities or schools to improve early detection and forecasting of SARS-CoV-2 transmission. Finally, the implementation of our surveillance system can guide preparedness efforts for future pandemics.

## Figures and Tables

**Table 1:** Average classification performance in terms of P(Outbreak|Class) (i.e., probability of outbreak (Yes/No) given the assigned risk class, L) and P(Class|Outbreak) (i.e., probability of the assigned risk class given the outbreak) at the low action-thresholds (predicted risk = 0.015).

Low Risk Group. P(Class = L) = 0.648			
P(Class = L|Outbreak = Yes)	P(Class ≠ L|Outbreak = Yes)	P(Class = L|Outbreak = No)	P(Class ≠ L|Outbreak = No)
0.23	0.77	0.73	0.27
P(Outbreak = Yes|Class = L)	P(Outbreak = No|Class = L)	P(Outbreak = Yes|Class ≠ L)	P(Outbreak = No|Class ≠ L)
0.06	0.94	0.37	0.63

**Table 2:** Average classification performance in terms of P(Outbreak|Class) (i.e., probability of outbreak (Yes/No) given the assigned risk class, H) and P(Class|Outbreak) (i.e., probability of the assigned risk class given the outbreak) at the high action-thresholds (predicted risk = 0.125).

High Risk Group P(Class = H) = 0.197			
P(Class = H|Outbreak = Yes)	P(Class ≠ H|Outbreak = Yes)	P(Class = H|Outbreak = No)	P(Class ≠ H|Outbreak = No)
0.51	0.49	0.14	0.86
P(Outbreak = Yes|Class = H)	P(Outbreak = No|Class = H)	P(Outbreak = Yes|Class ≠ H)	P(Outbreak = No|Class ≠ H)
0.40	0.60	0.09	0.91

**Table 3:** Average classification performance in terms of relative risk of COVID-19 outbreak by risk class. The relative risk is calculated as RR = $\frac{P(Outbreak=Yes|Class)}{P(Outbreak=Yes|Class=L)}$.

Risk Class	RR
L	−ref
M	3.45
H	5.95

## Data Availability

Open source datasets adopted for the study have been referenced throughout the manuscript. Restrictions apply to the availability of these data. Data was obtained from Fresenius Medical Care and may be available for specific, well-motivated requests, from the corresponding author with the permission of Fresenius Medical Care.

## Appendix A

**Table A1:** Variables included in the model.

Category	Variable	Reference Time
**Epidemic Status in the Country/Region (prefix: RG)**		
	cumulative cases	previous 7 days and last 7 days
	number of hospitalized	previous 7 days and last 7 days
	number of ICU patients	previous 7 days and last 7 days
	cumulative fatalities	previous 7 days and last 7 days
	cumulative recovered	previous 7 days and last 7 days
	trend of cumulative cases	last 7 days/previous 7 days
	trend of hospitalized patients	last 7 days/previous 7 days
	trend of ICU patients	last 7 days/previous 7 days
	trend of cumulative recovered in the last week	last 7 days/previous 7 days
	trend of cumulative fatalities	last 7 days/previous 7 days
**epidemic status in the clinic (prefix: CL)**		
	number of suspected COVID-19 cases	previous 14 days, previous 7 days, and last 7 days
	change in suspected cases	last 7 days–previous 7 days
	change in suspected cases	last 14 days–previous 14 days
**distance-weighted information of the adjacent clinics (prefix: CL)**		
	number of COVID-19 suspected cases in the closest clinics	previous 14d, previous 7 days, and last 7 days
	change in COVID-19 suspected cases in the closest clinics	last 7 days–previous 7 days
	change in COVID-19 suspected cases in the closest clinics	last 14 days–previous 14 days
**other parameters related to the clinic (prefix: CL)**		
	change in the number of treated patients	last 28 days–last 14 days
	change in the number of treatments	last 28 days–last 14 days
	change in the weekly dialysis frequency per clinic	last 28 days–last 14 days
	change in the weekly dialysis frequency per patient	last 28 days–last 14 days
	change in the number of treatments with pre/post-BT	last 28 days–last 14 days
	change in the number of treatments with pre/post-BT > 37 °C	last 28 days–last 14 days
	change in the percentage of treatments with pre/post-BT > 37 °C	last 28 days–last 14 days
	change in the mean value of pre/post-dialysis BT	last 28 days–last 14 days
	change in the number of treatments with pre-dyalisis diastolic BP	last 28 days–last 14 days
	change in the mean value of pre-dialysis diastolic BP	last 28 days–last 14 days
	change in the number of treatments with dialysis time	last 28 days–last 14 days
	change in the mean value of dialysis time	last 28 days–last 14 days
	change in the number of treatments with IDWG	last 28 days–last 14 days
	change in the mean value of IDWG	last 28 days–last 14 days
	change in the number of treatments with O2 sat	last 28 days–last 14 days
	change in the mean value of O2 sat	last 28 days–last 14 days
	change in the number of patients with lab tests	last 28 days–last 14 days
	change in the number of lab tests	last 28 days–last 14 days
	change in the number of lab tests with Albumin	last 28 days–last 14 days
	change in the mean value of Albumin	last 28 days–last 14 days
	change in the number of lab tests with lymphocytes	last 28 days–last 14 days
	change in the mean value of lymphocytes	last 28 days–last 14 days
	change in the number of lab tests with monocytes	last 28 days–last 14 days
	change in the mean value of monocytes	last 28 days–last 14 days
	change in the number of lab tests with neutrophils	last 28 days–last 14 days
	change in the mean value of neutrophils	last 28 days–last 14 days
	change in the number of lab tests with platelets	last 28 days–last 14 days
	change in the mean value of platelets	last 28 days–last 14 days
	change in the number of lab tests with PDW	last 28 days–last 14 days
	change in the mean value of PDW	last 28 days–last 14 days
	change in the number of lab tests with leukocytes	last 28 days–last 14 days
	change in the mean value of leukocytes	last 28 days–last 14 days
	change in the number of lab tests with D-dimer	last 28 days–last 14 days
	change in the mean value of D-dimer	last 28 days–last 14 days
	change in the number of lab tests with CRP	last 28 days–last 14 days
	change in the mean value of CRP	last 28 days–last 14 days
	change in the number of lab tests with IL-6	last 28 days–last 14 days
	change in the mean value of IL-6	last 28 days–last 14 days
	change in the number of lab tests with ANP	last 28 days–last 14 days
	change in the mean value of ANP	last 28 days–last 14 days
	change in the number of lab tests with BNP	last 28 days–last 14 days
	change in the mean value of BNP	last 28 days–last 14 days
	change in the number of lab tests with Ferritin	last 28 days–last 14 days
	change in the mean value of Ferritin	last 28 days–last 14 days
	Number of patients with at least one hospitalization	last 14 days
	Number of hospitalizations	last 14 days
